# Superiority of magnesium and vitamin B6 over magnesium alone on severe stress in healthy adults with low magnesemia: A randomized, single-blind clinical trial

**DOI:** 10.1371/journal.pone.0208454

**Published:** 2018-12-18

**Authors:** Etienne Pouteau, Marmar Kabir-Ahmadi, Lionel Noah, Andre Mazur, Louise Dye, Juliane Hellhammer, Gisele Pickering, Claude Dubray

**Affiliations:** 1 Nutritionals, Sanofi, Gentilly, France; 2 Statistics, Sanofi, Gentilly, France; 3 Unité de Nutrition Humaine, INRA, Université Clermont Auvergne, Clermont-Ferrand, France; 4 Nutrition and Behaviour Group, School of Psychology, University of Leeds, Leeds, United Kingdom; 5 Contract Research and Saliva Lab, Daacro, Trier, Germany; 6 Clinical Pharmacology Department, CHU, Université Clermont Auvergne, Clermont-Ferrand, France; Indiana University Richard M Fairbanks School of Public Health, UNITED STATES

## Abstract

**Introduction:**

Animal and clinical studies suggest complementary effects of magnesium and high-dose pyridoxine (vitamin B6) on stress reduction. This is the first randomized trial evaluating the effects of combined magnesium and vitamin B6 supplementation on stress in a stressed population with low magnesemia using a validated measure of perceived stress.

**Methods:**

In this Phase IV, investigator-blinded trial (EudraCT: 2015-003749-24), healthy adults with Depression Anxiety Stress Scales (DASS-42) stress subscale score >18 and serum magnesium concentration 0.45 mmol/L–0.85 mmol/L, were randomized 1:1 to magnesium–vitamin B6 combination (Magne B6 [Mg–vitamin B6]; daily dose 300 mg and 30 mg, respectively) or magnesium alone (Magnespasmyl [Mg]; daily dose 300 mg). Outcomes included change in DASS-42 stress subscale score from baseline to Week 8 (primary endpoint) and Week 4, and incidence of adverse events (AEs).

**Results:**

In the modified intention-to-treat analysis (N = 264 subjects), both treatment arms substantially reduced DASS-42 stress subscale score from baseline to Week 8 (Mg–vitamin B6, 44.9%; Mg 42.4%); no statistical difference between arms was observed (p>0.05). An interaction (p = 0.0097) between baseline stress level and treatment warranted subgroup analysis (as per statistical plan); adults with severe/extremely severe stress (DASS-42 stress subscale score ≥25; N = 162) had a 24% greater improvement with Mg–vitamin B6 versus Mg at Week 8 (3.16 points, 95% CI 0.50 to 5.82, p = 0.0203). Consistent results were observed in the per protocol analysis and at Week 4. Overall, 12.1% of Mg–vitamin B6 treated and 17.4% of Mg-treated subjects experienced AEs potentially treatment related.

**Conclusions:**

These findings suggest oral Mg supplementation alleviated stress in healthy adults with low magnesemia and the addition of vitamin B6 to Mg was not superior to Mg supplementation alone. With regard to subjects with severe/extremely severe stress, this study provides clinical support for greater benefit of Mg combined with vitamin B6.

## Introduction

Magnesium is the second most abundant intracellular cation after potassium [1, 2]. It plays an essential physiological role in the body as an enzymatic cofactor in over 600 biochemical reactions [1]. The physiological impact of stress on intracellular and extracellular magnesium concentrations has been well described [3, 4]. Hormones released during stress, including catecholamines and corticosteroids, have been shown to enhance a shift of magnesium from the intracellular to the extracellular space, leading to increased urinary excretion of magnesium and subsequent decrease in serum magnesium concentrations [3, 5]. In turn, low serum magnesium concentrations increase the release of stress-associated hormones including catecholamines, adrenocorticotrophic hormone and cortisol in response to stress, and affect their access to the brain, creating a vicious circle of reduced resistance to stress and further magnesium depletion [4, 6].

The relationship between serum magnesium concentration and stress has been evidenced in a clinical trial that reported an association between low serum magnesium concentrations and greater perceived stress in otherwise healthy women [7]. Other studies have documented a positive effect of magnesium supplementation on symptoms and biomarkers of stress. In a double-blind, randomized trial of 46 healthy adults aged 60–75 years, magnesium supplementation (magnesium 500 mg per day administered as magnesium oxide tablets for 8 weeks) improved subjective measures of insomnia, which is recognized as a symptom of stress [8, 9]. Magnesium supplementation over a period of one month (magnesium 500 mg per day in a magnesium oxide tablet) has also been shown to significantly decrease basal serum cortisol concentrations, a biomarker of stress, in students [10].

High-dose (100–300 mg daily) pyridoxine (vitamin B6) has also been proposed as an anti-stress therapy; vitamin B6 exerts modulatory effects on neurotransmitters that affect depression and anxiety, and may reduce blood pressure and act peripherally to reduce the physiological impact of corticosteroid release [11]. In rodent studies, high-dose vitamin B6 was able to correct low serum and tissue magnesium concentrations induced by dietary magnesium depletion and prevent stress-induced gastric ulcers [12–14]. One proposed mechanism is that vitamin B6 facilitates cellular uptake of magnesium, which both limits excretion and increases its effectiveness (since the mineral is primarily an intracellular cation) [15, 16]. In light of the direct roles of magnesium and vitamin B6 in the modulation of stress and associated pathways, as well as their complementary effects, examination of the efficacy of magnesium and concomitant vitamin B6 supplementation in individuals with low concentrations of magnesium is warranted. However, as recently reviewed, no randomized clinical trial to date has investigated the efficacy of magnesium plus vitamin B6 supplementation on stress in such a population using a validated measure of perceived stress as an outcome [17].

A combination of magnesium lactate dehydrate and pyridoxine hydrochloride in a 10:1 ratio (magnesium lactate dehydrate 300 mg/pyridoxine hydrochloride 30 mg) is available as an over-the-counter supplement (e.g. Magne B6), and is indicated for the prevention and treatment of magnesium deficiency and associated symptoms (including fatigue, mild anxiety, and nervousness) (Magne B6 SmPC) [18]. This specific combination of magnesium and vitamin B6 in a 10:1 ratio has been shown to provide faster relief of magnesium-deficiency symptoms than magnesium alone in magnesium-deficient animals [12]. The objective of the current trial was to compare this magnesium–vitamin B6 combination versus magnesium alone in stressed healthy adults with suboptimal serum magnesium concentrations using the stress subscale of the validated Depression Anxiety Stress Scales (DASS-42) self-assessment tool [19].

## Methods

### Trial design

**Fig 1:**
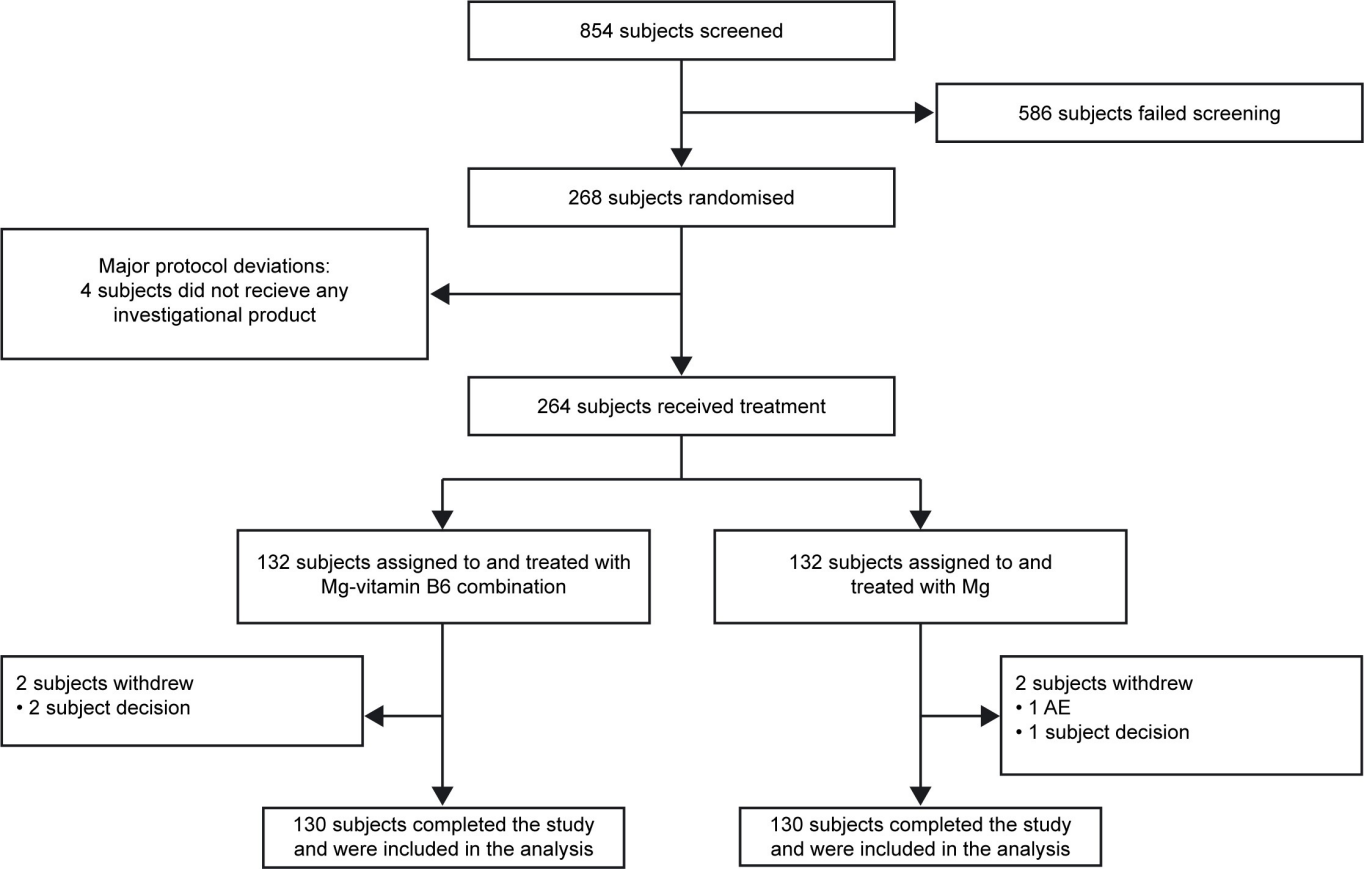
CONSORT flowchart. AE, adverse event; Mg, magnesium.

**Fig 2:**
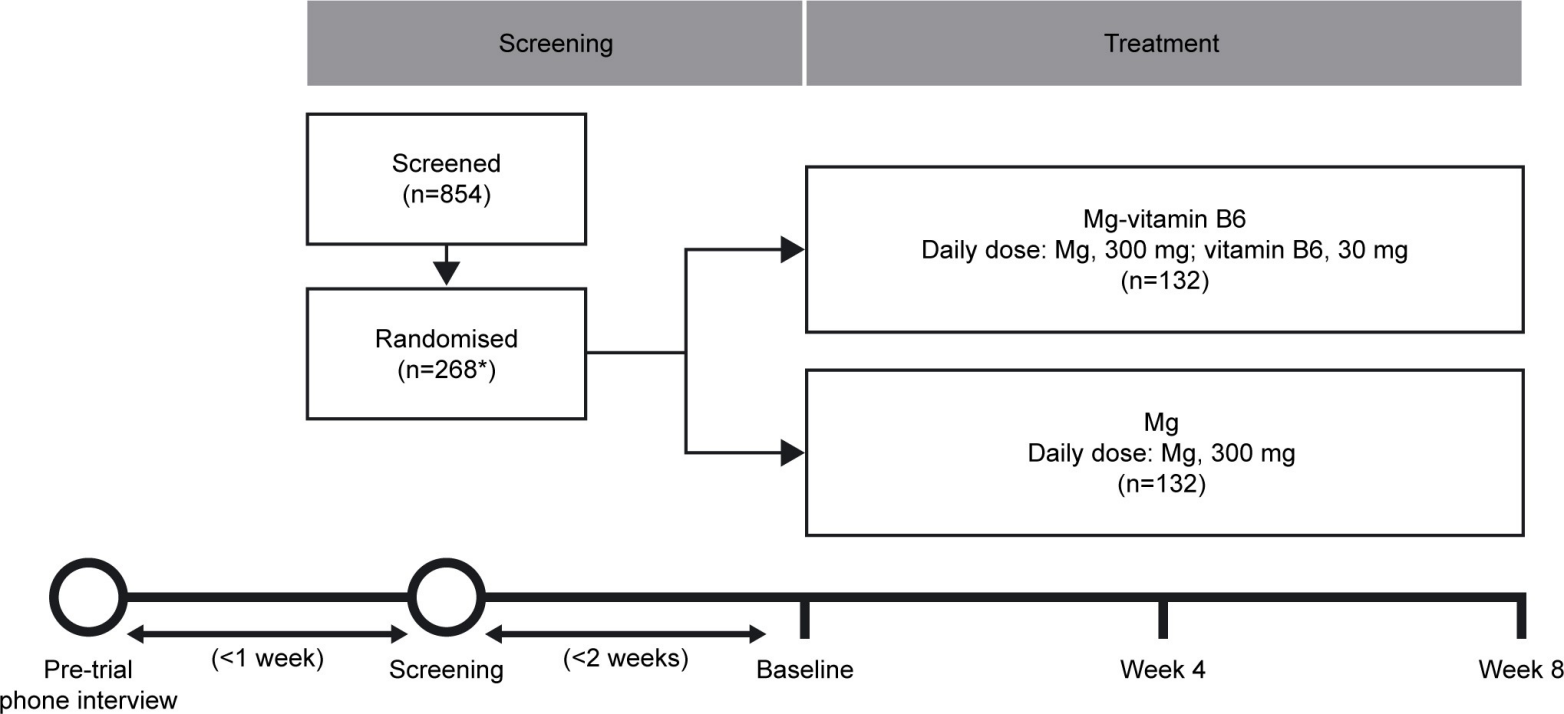
Trial design. *Four subjects did not proceed to the treatment phase and did not receive any investigational product due to major protocol deviations. Mg, magnesium.

This was an 8-week, Phase IV, randomized, controlled, investigator-blinded, parallel-group trial stratified by sex (EudraCT Number: 2015-003749-24) (**Fig 1**). The trial was carried out at 4 clinical trial centers in France. Healthy subjects completed a pre-trial telephone interview within 1 week prior to screening; the baseline visit took place <2 weeks after the screening visit (**Fig 2**). The 8-week treatment period comprised visits at Week 4 and Week 8. Subjects were randomized 1:1 to treatment with either the magnesium–vitamin B6 (Mg–vitamin B6) combination or magnesium (Mg) alone. Subjects were randomly divided at baseline in order to avoid systematic differences with respect to known or unknown variables that could affect outcomes.

### Standard protocol approvals, registration and participant consents

The protocol complied with recommendations of the Declaration of Helsinki, amended by the 64th World Medical Association General Assembly, Fortaleza, Brazil, October 2013, and the International Conference on Harmonization (ICH) guidelines for good clinical practice (GCP), all applicable laws, rules and regulations. The protocol also complied with the laws and regulations, as well as any applicable guidelines, from France, where the trial was conducted. Ethical approval was granted by The Ethical Committee (comité de protection des personnes) at Le Centre Hospitalier Universitaire (CHU), France. All subjects provided written informed consent.

### Subjects

Subjects were between 18 and 50 years of age with moderate to extremely severe stress at screening, defined as a DASS-42 stress subscale score of >18 [20]. Additionally, subjects must have presented with suboptimal serum magnesium concentrations (assessed locally at each of the trial sites), defined as serum magnesium concentrations between 0.45 mmol/L and 0.85 mmol/L; these were measured locally at the investigation centers. The upper limit of the serum magnesium cut-off was chosen based on previous work establishing an evidence-based reference interval (central 95th percentile) as 0.75 mmol/L to 0.95 mmol/L with a mean concentration of 0.85 mmol/L. The serum magnesium cut-off of 0.85 mmol/L has been previously determined as the lower limit adjusted to a value for health in a trial of over 15,000 subjects [21]. At screening, magnesium concentrations were assessed with blood serum samples. During the study (baseline, Week 4 and Week 8), magnesium concentrations were assessed in the erythrocytes; erythrocytes are thought to accurately represent magnesium cell content within the body and provide an accurate reflection of the whole body magnesium status. Additional inclusion criteria were, a body mass index (BMI) of >18.5 and ≤29.9 kg/m^2^, and the use of an effective method of contraception during the trial period for female subjects. Key exclusion criteria included: exposure to therapies prohibited by the protocol (including levodopa, quinidine, and proton-pump inhibitors) within 3 months prior to screening; concomitant conditions or diseases that could make subjects non-evaluable for the primary endpoint; severe hypomagnesemia (defined as serum magnesium of ≤0.45 mmol/L) [21]; participant-reported moderate or severe kidney failure; confirmed diagnosis of type 1 or 2 diabetes; any known addiction to drugs and alcohol; alcohol intake of ≥3 drinks per day.

### Interventions

Each participant received coated tablets of either Mg–vitamin B6 combination (Magne B6; 470 mg magnesium lactate dihydrate and 5 mg pyridoxine hydrochloride) or Mg alone (Magnespasmyl [Mg]; 465.4 mg magnesium lactate dihydrate). Tablets were self-administered orally, with subjects taking 6 coated tablets per day (corresponding to approximately 300 mg elemental magnesium with or without 30 mg vitamin B6) divided into 3 intakes (2 tablets during each meal [breakfast, lunch, and dinner]) over a period of 8 weeks.

Tablets were provided in treatment sets with 50 tablets, dispatched in 5 blister packs of 10 tablets each. Subjects received 4 treatment kits at randomization for the following 4 weeks, after which they received an additional 4 treatment kits at the Week 4 visit for use until the end of the trial.

### Objectives

The primary objective was to compare magnesium in combination with vitamin B6 supplementation versus magnesium alone on stress, evaluated by the stress subscale from the DASS-42 test, in healthy adults with stress and suboptimal serum magnesium concentrations. A secondary objective was to evaluate the safety profile of the Mg–vitamin B6 combination versus Mg alone, as determined by the incidence of adverse events (AEs).

### Outcome variables and assessments

The primary outcome variable was change in the DASS-42 stress subscale score from baseline to Week 8. A secondary outcome variable was change in the DASS-42 stress subscale score from baseline to Week 4. Depression and anxiety subscales of DASS-42 were also evaluated during the trial but these data will be reported in separate publications. Subjects completed the DASS-42 questionnaire at screening, baseline, Week 4 and Week 8.

The DASS-42 is a 42-item, clinically validated questionnaire that includes three subscales designed to measure negative emotional states of depression, anxiety and stress over the previous week [18]. The stress component of DASS-42 comprises 14 questions with a 4-point scale for self-reported scoring: 0 = did not apply to me at all; 1 = applied to me to some degree, or some of the time; 2 = applied to me a considerable degree, or a good part of the time; 3 = applied to me very much, or most of the time [19, 20].

Treatment adherence was tracked by counting dispensed and unused tablets at Week 4 and Week 8 visits. A participant was considered non-adherent if they did not dispense the planned dose as required by the protocol: i.e., 8 weeks of treatment; oral route; 6 coated tablets per day; 2 coated tablets to be swallowed during each meal.

Safety outcomes included incidence of AEs, treatment-related AEs, and serious adverse events (SAEs). AEs were coded according to the Medical Dictionary for Regulatory Activities (MedDRA), version 20.0.

### Sample size

An overall sample size of 268 (134 in each treatment arm) was selected to obtain 119 evaluable subjects per treatment arm (assuming 10% of subjects would be non-evaluable), and achieve 80% power to detect a difference of 3 points on the DASS-42 stress subscale score change from baseline at Week 4 and Week 8 between intervention arms (delta of superiority corresponding to ~15% of baseline value, which was expected to be ~20). Group standard deviations (SD) of 8 points were estimated with a significance level (alpha) of 0.05 using a two-sided equal-variance t-test and a Mann–Whitney test, and assuming normal distribution.

The clinically relevant difference of 3 points was defined with expert clinicians. The SD estimation was based on the evaluation of DASS-42 stress subscale score of “emailed standardized cognitive behavioral treatment of work-related stress” [22].

### Randomization and blinding

The randomization sequence was centralized and generated automatically under the responsibility of the French Clinical Study Unit of Sanofi using validated software (SAS 9.2), and treatments were packaged according to this sequence. At randomization, the trial site contacted an Interactive Web Response System to allocate treatment kits to subjects. Treatment kits were indistinguishable and labelled with randomized treatment kit numbers. Investigators were blinded by ensuring that treatment dispensing and treatment return were handled separately.

### Statistical methods

The modified Intention-To-Treat (mITT) population was defined as all subjects with an evaluable DASS-42 stress subscale score at baseline and at least one other time point during the treatment period. The Per Protocol (PP) population was defined as all subjects included in the mITT analysis without any major protocol deviations.

Of note, the mITT population comprised some subjects who had a baseline DASS-42 stress subscale score ≤18. This can be attributed to variations in DASS-42 stress subscale score between screening and baseline (~two weeks occurred between screening and baseline). Therefore, the statistical analysis plan was amended after the database lock based on a decision from the scientific committee, such that the PP population should include all subjects in the mITT without any major protocol violations, and with a DASS-42 stress subscale score >18 at baseline.

The population included in the Safety Set (SS) comprised all subjects included in the trial with at least one consumption of trial product and was used for safety evaluations.

Differences between treatment arms in change from baseline to Week 4 or Week 8 in DASS-42 stress subscale scale score were calculated by means of a repeated measures analysis of covariance (ANCOVA), with DASS-42 stress subscale score at baseline and stratification factor (sex) as covariates, and visit and interaction between visit and treatment group and interaction between baseline value and treatment group as fixed effects.

Adjusted mean was calculated from model ANCOVA adjusted by baseline value of DASS-42 and the interaction of baseline and treatment.

### Subgroup analyses

Based on European Medical Agency (EMA) guidelines for the adjustment of baseline covariates [23], the statistical analysis plan was amended after the blinded database lock to include subgroup analyses of primary and secondary efficacy endpoints by baseline stress level, as a significant interaction (p < 0.05) was present between baseline DASS-42 stress subscale scores and treatment (assessed firstly as a continuous variable and then categorically by class [‘normal and moderate stress’ versus ‘severe and extremely severe stress’]).

## Results

### Participant disposition

Between May 2016 and January 2017, 854 subjects were screened and 268 were enrolled (134 in each treatment arm) (**Fig 1**). Two subjects in each treatment arm received no trial treatment, and were excluded from the mITT and PP analysis. Of note, 26 subjects (13 in each arm) in the mITT analysis had baseline DASS-42 stress subscale scores ≤18 despite the inclusion criteria used at screening. This was due to variation in stress levels between screening and baseline. These 26 subjects were excluded from the PP analysis in line with statistical analysis plan, in addition to a further five subjects (two in the Mg–vitamin B6 combination arm and three in the Mg alone arm) who were excluded due to an observed treatment adherence of ≤75%, defined as a major protocol deviation. For the subgroup analyses specified in the results, the 26 subjects with baseline DASS-42 stress subscale scores ≤18 were included in the mITT but excluded from the PP analysis. Overall, 260 subjects (130 in each arm) completed the trial (**Fig 1**).

### Baseline demographics and characteristics

Mean (SD) age at baseline was 31.6 (8.5) years, and mean BMI was 23.0 kg/m^2^. The majority (74%) of subjects were female. Demographic characteristics were similar across treatment arms (**Table 1**). The distribution of subjects across DASS-42 stress subscale levels was also similar in each treatment arm, with approximately 60% in each group classified as having severe or extremely severe stress (**Table 2**). There was no association between DASS-42 score and overweight (25–<30 kg/m^2^) status (mean [SD] DASS-42 for those not overweight versus overweight: 27.9 [7.1] versus 26.9 [7.0]; p > 0.05).

**Table 1:** Participant demographic and disease characteristics at baseline (mITT population).

Parameter	Mg–vit B6 combination (N = 132)	Mg (N = 132)	Total (N = 264)
Age (years), mean (SD)	31.2 (8.4)	32.1 (8.6)	31.6 (8.5)
Sex female, n (%)	98 (74.2)	97 (73.5)	195 (73.9)
BMI, kg/m^2^, mean (SD)	23.0 (3.0)	22.9 (2.7)	23.0 (2.8)
BMI category, n (%)			
Normal (18.5–<25 kg/m^2^)	101 (76.5)	102 (77.3)	203 (76.9)
Overweight (25–<30 kg/m^2^)	31 (23.5)	30 (22.7)	61 (23.1)
Systolic blood pressure (mmHg), mean (SD)	118.2 (13.4)	116.7 (11.1)	117.4 (12.3)
Diastolic blood pressure (mmHg), mean (SD)	73.9 (9.1)	72.8 (8.3)	73.4 (8.7)
Heart rate (bpm), mean (SD)	68.1 (12.8)	67.6 (11.6)	67.9 (12.2)
Serum Mg^a^ (mmol/L)			
Mean (SD)	0.80 (0.0)	0.80 (0.0)	0.80 (0.04)
Median (Min, Max)	0.80 (0.7, 0.8)	0.80 (0.7, 0.8)	0.82 (0.66, 0.84)
Erythrocyte Mg (mmol/L), mean (SD)	1.80 (0.30)	1.80 (0.40)	1.83 (0.31)
Serum B6 (nmol/L), mean (SD)	50.6 (68.8)	46.5 (27.6)	48.6 (52.3)
DASS-42 stress subscale score, mean (SD)	27.7 (7.3)	27.6 (7.0)	27.7 (7.1)
DASS-42 total score, mean (SD)	58.3 (21.3)	58.4 (20.9)	58.4 (20.9)

^a^At screening visit;

BMI, body mass index; bpm, beats per minute; DASS, Depression Anxiety Stress Scales; mITT, modified intention-to-treat; Mg, magnesium; SD, standard deviation; Vit B6, vitamin B6.

**Table 2:** Distribution of DASS-42 stress subscale scores at baseline and Week 8 (mITT population).

		Baseline			Week 8	
DASS-42 stress score	Mg–vit B6 combination (N = 132)	Mg (N = 132)	Total (N = 264)	Mg–vit B6 combination (N = 132)	Mg (N = 132)	Total (N = 264)
Normal (score^a^ 0–14), n (%)	4 (3.0)	6 (4.5)	10 (3.8)	75 (57.3)	69 (53.1)	144 (55.2)
Mild (score^a^ 15–18), n (%)	9 (6.8)	7 (5.3)	16 (6.1)	18 (13.7)	21 (16.2)	39 (14.9)
Moderate (score^a^ 19–25), n (%)	41 (31.1)	35 (26.5)	76 (28.8)	29 (22.1)	17 (13.1)	46 (17.6)
Severe (score^a^ 26–33), n (%)	46 (34.8)	59 (44.7)	105 (39.8)	7 (5.3)	15 (11.5)	22 (8.4)
Extremely severe (score^a^ 34–42), n (%)	32 (24.2)	25 (18.9)	57 (21.6)	2 (1.5)	8 (6.2)	10 (3.8)

^a^Stress subscale score

DASS, Depression Anxiety Stress Scale; mITT, modified intention-to-treat; Mg, magnesium; Vit B6, vitamin B6.

Overall, 39% of subjects in the Mg–vitamin B6 combination arm and 40% in the Mg arm reported at least one medication prior to trial entry, 39% in both arms had ongoing medication at baseline, and 25% and 21%, respectively, started at least one medication between baseline and Week 8. None of the prior medications have any known interaction with the trial product or effect on stress.

### Treatment adherence

Between baseline randomization and Week 8, mean (SD) assumed treatment adherence was 94% (7.1) in the Mg–vitamin B6 combination arm and 93.0% (9.2) in the Mg arm; 96% of subjects (127/132) in each arm had an assumed treatment adherence of ≥80%.

### Efficacy

#### Overall change from baseline in DASS-42 stress subscale score

In the mITT population, both treatments reduced DASS-42 stress subscale score from baseline to Week 8, reducing the overall proportion of subjects with severe or extremely severe stress at baseline from approximately 60% to approximately 12% (**Table 2**). The DASS-42 stress subscale score improved by 44.9% from a mean (SD) of 27.7 (7.3) points at baseline to 14.5 (7.4) points at Week 8 in the Mg–vitamin B6 combination arm, representing a change in adjusted mean of −12.44 points (95% confidence interval [CI] −13.83 to −11.05). Scores also improved in the Mg group, by 42.4%, from 27.6 (7.0) points at baseline to 15.3 (9.5) points at Week 8, with a change in adjusted mean of −11.72 points (95% CI −13.10 to −10.33). The difference between treatment arms was not statistically significant (0.72 points, 95% CI −1.15 to 2.59, p > 0.05) (**Table 3**). Similar findings were observed in the PP population with no statistically significant difference between treatment arms (1.06 points, 95% CI −0.99 to 3.10, p > 0.05 (**Table 3**).

**Table 3:** Change in DASS-42 stress subscale score from baseline to Week 4 and to Week 8.

**mITT population**	**Mg–vit B6 combination (N = 132)**	**Mg (N = 132)**
Change from baseline to Week 4^a^ (95% CI)	−8.94 (−10.22 to −7.65)	−7.58 (−8.86 to −6.30)
Difference between treatment arms	1.35 (−0.36 to 3.06), p = 0.1203	
Change from baseline to Week 8^a^ (95% CI)	−12.44 (−13.83 to −11.05)	−11.72 (−13.10 to −10.33)
Difference between treatment arms	0.72 (−1.15 to 2.59), p = 0.4472	
**PP population**	**Mg–vit B6 combination (N = 117)**	**Mg (N = 116)**
Change from baseline to Week 4^a^^b^ (95% CI)	−9.59 (−11.03 to −8.15)	−8.04 (−9.45 to −6.63)
Difference between treatment arms	1.55 (−0.33 to 3.43), p = 0.1056	
Change from baseline to Week 8^a^^b^ (95% CI)	−13.26 (−14.81 to −11.71)	−12.21 (−13.73 to −10.68)
Difference between treatment arms	1.06 (−0.99 to 3.10), p = 0.3095	

^a^Difference from baseline in adjusted mean.

^**b**^Subjects with subscale scores ≤18 baseline were excluded from the PP population.

CI, confidence interval; DASS, Depression Anxiety Stress Scale; mITT, modified intention-to-treat; Mg, magnesium; PP, per protocol; Vit B6, vitamin B6.

In the mITT population, both treatment arms improved DASS-stress subscale score from baseline to Week 4 (Mg–vitamin B6, −8.94 points, 95% CI −10.22 to −7.65; Mg, −7.58, 95% CI −8.86 to −6.30); the difference between treatment arms was not statistically significant (1.35 points, 95% CI −0.36 to 3.06, p > 0.05) (**Table 3**). Similar improvements from baseline to Week 4 were observed in the PP population, with no statistically significant difference between treatment arms (1.55 points, 95% CI −0.33 to 3.43, p > 0.05; **Table 3**).

#### Change in DASS-42 stress subscale score in baseline stress severity subgroups

According to recent EMA guidelines, the identification of heterogeneous benefits within samples warrants additional subgroup analyses [23], thus a subgroup analysis was performed, in line with the statistical analysis plan. The interaction test to assess association between DASS-42 stress subscale score at baseline and treatment showed a statistically significant association in both the mITT population (p = 0.0097) and the PP population (p = 0.0171). Therefore, an analysis of change in DASS-42 stress subscale score by subgroup according to baseline score was carried out in both the mITT and PP populations.

**Fig 3:**
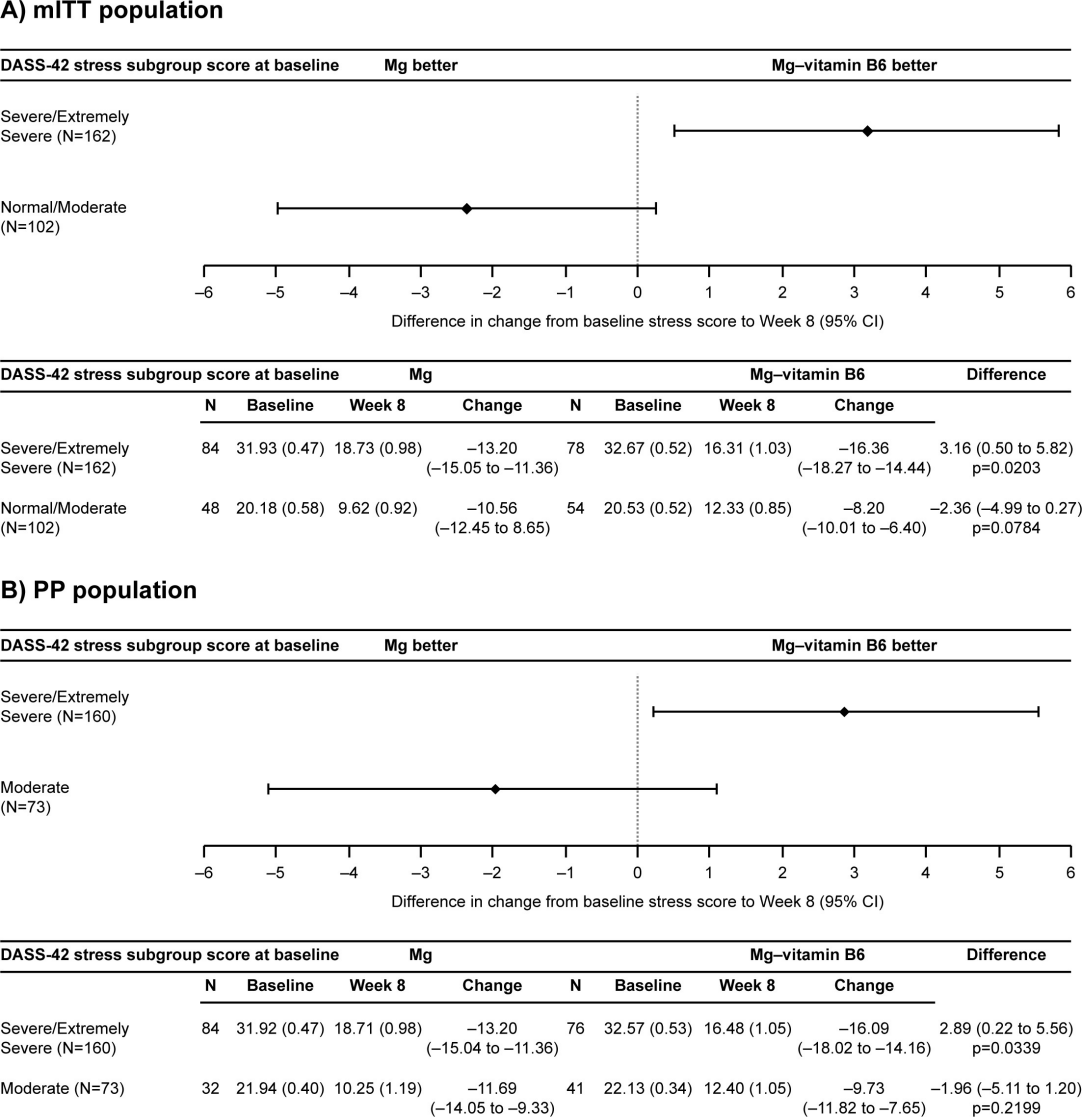
**Change in DASS-42 stress subscale score from baseline to Week 8 in the mITT (A) and PP (B) subgroup populations.** CI, confidence interval; DASS, Depression Anxiety Stress Scale; Mg, magnesium; mITT, modified intention-to-treat; PP, per protocol; Vit B6, vitamin B6.

For mITT population, the subgroups were defined as normal to moderate stress and severe to extremely severe stress; in the PP population the subgroups were defined as moderate stress and extreme to extremely severe stress. The severe to extremely severe stress subgroup comprised subjects with baseline DASS-42 stress subscale scores between 26 and 42 (mITT and PP); the normal to moderate stress subgroup comprised subjects with baseline stress subscale scores between 0 and 25 (mITT) [20]; the moderate stress subgroup comprised subjects with scores between 19 and 25 and did not include subjects with baseline DASS-42 stress subscale scores ≤18, who were already excluded from the PP as per the statistical analysis plan. For both mITT and PP population analyses, baseline DASS-42 stress subscale scores were similar between treatment arms in the severe and extremely severe stress subgroup (mITT: Mg–vitamin B6 combination, n = 78; Mg, n = 84 and PP: Mg–vitamin B6 combination, n = 76; Mg n = 84) (**Fig 3**) and the normal to moderate (mITT population) and moderate (PP population) stress subgroups (mITT: Mg–vitamin B6 combination, n = 54; Mg, n = 48 and PP; Mg–vitamin B6 combination n = 41; Mg, n = 32) (**Fig 3**).

In the mITT population, both treatment arms in the severe and extremely severe stress subgroup had an improvement in DASS-42 subscale score from baseline to Week 8. The Mg–vitamin B6 arm had a 50.1% improvement (−16.36 points, 95% CI, −18.27 to −14.44) and the Mg arm had a 41.3% improvement (−13.20 points, 95% CI, −15.05 to −11.36). There was a statistically significant improvement from baseline to Week 8 in Mg–vitamin B6-treated subjects compared with the Mg group (**Fig 3A**). The improvement was 23.9% greater (3.16 points, 95% CI 0.50 to 5.82, p = 0.0203) for the Mg–vitamin B6 combination group versus the Mg group. There was no significant difference between treatment arms in subjects with normal to moderate stress (−2.36 points, 95% CI −4.99 to 0.27, p > 0.05) (**Fig 3A**).

Similar results were observed in the PP population (**Fig 3B**). In subjects with severe or extremely severe stress, both treatment arms improved DASS-42 stress subscale score. The Mg–vitamin B6 arm had an improvement of 49.4% (−16.09 points, 95% CI −18.02 to −14.16) and the Mg arm had an improvement of 41.3% (−13.20 points, 95% CI−15.04 to −11.36). A statistically significant 21.9% (2.89 points, 95% CI 0.22 to 5.56, p = 0.0339) greater improvement from baseline to Week 8 was observed in Mg–vitamin B6-treated versus Mg-treated subjects. There was no significant difference between treatment arms in subjects with moderate stress (−1.96 points; 95% CI −5.11 to 1.20, p > 0.05).

Changes from baseline to Week 4 are shown in **Table 4**. In the mITT population, in subjects with severe or extremely severe stress, the improvement from baseline was 38.2% (3.37 points; 95% CI 1.02 to 5.73) greater with Mg–vitamin B6 combination compared with Mg, and the difference in change between treatments was statistically significant (p = 0.0053). In subjects with normal to moderate stress, the change from baseline was not significantly different between treatment arms (−1.22 points; 95% CI −3.73 to 1.29; p > 0.05).

**Table 4:** Change in DASS-42 stress subscale score from baseline to Week 4 by DASS-42 subscale subgroup score at baseline.

**mITT population**	**Severe to extremely severe stress (N = 162)**		**Normal to moderate stress (N = 102)**	
	**Mg–vit B6 combination (N = 78)**	**Mg (N = 84)**	**Mg–vit B6 combination (N = 54)**	**Mg (N = 48)**
Baseline, adjusted mean (SE)	32.67 (0.52)	31.93 (0.47)	20.53 (0.52)	20.18 (0.58)
Week 4, adjusted mean (SE)	20.47 (0.96)	23.10 (0.91)	14.90 (0.87)	13.33 (0.94)
Change in adjusted mean (95% CI)	−12.21 (−13.90 to −10.51)	−8.83 (−10.47 to −7.20)	−5.63 (−7.35 to −3.91)	−6.85 (−8.68 to −5.03)
Difference in change in adjusted mean between arms (95% CI)	3.37 (1.02 to 5.73), p = 0.0053		−1.22 (−3.73 to 1.29), p = 0.3354	
**PP population**	**Severe to extremely severe stress (N = 160)**		**Moderate stress (N = 73)**	
	**Mg–vit B6 combination (N = 76)**	**Mg (N = 84)**	**Mg–vit B6 combination (N = 41)**	**Mg (N = 32)**
Baseline, adjusted mean (SE)	32.57 (0.53)	31.92 (0.47)	22.13 (0.34)	21.94 (0.40)
Week 4, adjusted mean (SE)	20.25 (0.97)	23.08 (0.91)	15.88 (1.04)	13.90 (1.18)
Change in adjusted mean (95% CI)	−12.33 (−14.04 to −10.61)	−8.83 (−10.46 to −7.20)	−6.24 (−8.30 to −4.19)	−8.03 (−10.36 to −5.70)
Difference in change in adjusted mean between arms (95% CI)	3.50 (1.13 to 5.86), p = 0.0041		−1.79 (−4.89 to 1.32), p = 0.2550	

CI, confidence interval; DASS, Depression Anxiety Stress Scale; mITT, modified intention-to-treat; Mg, magnesium; PP, strict per protocol analysis; SE, standard error; Vit B6, vitamin B6. Standard error rather than standard deviation was calculated for subgroups to account for the means being adjusted.

Change from baseline to Week 4 in the PP population was consistent with the mITT population findings (**Table 4**). In subjects with severe or extremely severe stress, the improvement from baseline was 39.6% (3.50 points, 95% CI 1.13 to 5.86) greater with the Mg–vitamin B6 combination compared with Mg, and the difference was statistically significant (p = 0.0041). In subjects with moderate stress, there was no significant difference between treatment arms (−1.79 points, 95% CI −4.89 to 1.32; p > 0.05).

### Safety

Overall, 41% (54/132) of subjects in the Mg–vitamin B6 combination arm and 40% (53/132) in the Mg arm experienced at least one AE. In total, 12% (16/132) of subjects in the Mg–vitamin B6 combination group and 17% (23/132) in the Mg group experienced at least one AE considered possibly related to trial treatment, as assessed by the trial physician. The most common treatment-related AE was diarrhea, reported by 4.5% (6/132) of subjects in the Mg–vitamin B6 combination arm and 7.6% (10/132) in the Mg arm (**Table 5**). One (0.8%) subject in the Mg arm had at least one AE of severe intensity (gastroenteritis); no such events occurred in the Mg–vitamin B6 combination arm.

**Table 5:** Treatment-related adverse events occurring in ≥1% of the total population (SS).

System Organ Class, Preferred Term	Mg–vit B6 combination (N = 132)		Mg (N = 132)		Total (N = 264)	
	Subjects with ≥1 TRAE, n (%)	TRAEs, n (%)	Subjects with ≥1 TRAE, n (%)	TRAEs, n (%)	Subjects with ≥1 TRAE, n (%)	TRAEs, n (%)
**Total**	16 (12.12)	26 (100)	23 (17.42)	43 (100)	39 (14.77)	69 (100)
**Gastrointestinal disorders**	11 (8.33)	18 (69.23)	18 (13.64)	36 (83.72)	29 (10.98)	54 (78.26)
Abdominal discomfort	1 (0.76)	1 (3.85)	0 (0.00)	0 (0.00)	1 (0.38)	1 (1.45)
Abdominal distension	2 (1.52)	2 (7.69)	0 (0.00)	0 (0.00)	2 (0.76)	2 (2.90)
Abdominal pain	3 (2.27)	3 (11.54)	6 (4.55)	10 (23.26)	9 (3.41)	13 (18.84)
Abdominal pain upper	0 (0.00)	0 (0.00)	2 (1.52)	2 (4.65)	2 (0.76)	2 (2.90)
Constipation	0 (0.00)	0 (0.00)	1 (0.76)	1 (2.33)	1 (0.38)	1 (1.45)
Diarrhea	6 (4.55)	6 (23.08)	10 (7.58)	19 (44.19)	16 (6.06)	25 (36.23)
Dry mouth	1 (0.76)	1 (3.85)	0 (0.00)	0 (0.00)	1 (0.38)	1 (1.45)
Dysphagia	1 (0.76)	1 (3.85)	0 (0.00)	0 (0.00)	1 (0.38)	1 (1.45)
Feces soft	3 (2.27)	3 (11.54)	2 (1.52)	2 (4.65)	5 (1.89)	5 (7.25)
Flatulence	0 (0.00)	0 (0.00)	1 (0.76)	1 (2.33)	1 (0.38)	1 (1.45)
Frequent bowel movements	1 (0.76)	1 (3.85)	0 (0.00)	0 (0.00)	1 (0.38)	1 (1.45)
Nausea	0 (0.00)	0 (0.00)	1 (0.76)	1 (2.33)	1 (0.38)	1 (1.45)
**General disorders and administration site conditions**	1 (0.76)	1 (3.85)	0 (0.00)	0 (0.00)	1 (0.38)	1 (1.45)
Asthenia	1 (0.76)	1 (3.85)	0 (0.00)	0 (0.00)	1 (0.38)	1 (1.45)
**Metabolism and nutrition disorders**	0 (0.00)	0 (0.00)	1 (0.76)	1 (2.33)	1 (0.38)	1 (1.45)
Increased appetite	0 (0.00)	0 (0.00)	1 (0.76)	1 (2.33)	1 (0.38)	1 (1.45)
**Nervous system disorders**	2 (1.52)	2 (7.69)	5 (3.79)	5 (11.63)	7 (2.65)	7 (10.14)
Dizziness	0 (0.00)	0 (0.00)	1 (0.76)	1 (2.33)	1 (0.38)	1 (1.45)
Headache	2 (1.52)	2 (7.69)	3 (2.27)	3 (6.98)	5 (1.89)	5 (7.25)
Hypersomnia	0 (0.00)	0 (0.00)	1 (0.76)	1 (2.33)	1 (0.38)	1 (1.45)
**Psychiatric disorders**	2 (1.52)	2 (7.69)	0 (0.00)	0 (0.00)	2 (0.76)	2 (2.90)
Sleep disorder	2 (1.52)	2 (7.69)	0 (0.00)	0 (0.00)	2 (0.76)	2 (2.90)
**Skin and subcutaneous tissue disorders**	3 (2.27)	3 (11.54)	1 (0.76)	1 (2.33)	4 (1.52)	4 (5.80)
Dermatitis acneiform	1 (0.76)	1 (3.85)	1 (0.76)	1 (2.33)	2 (0.76)	2 (2.90)
Rash papular	2 (1.52)	2 (7.69)	0 (0.00)	0 (0.00)	2 (0.76)	2 (2.90)

Mg, magnesium; SS, safety set; TRAE, treatment-related adverse event; Vit B6, vitamin B6.

Treatment dose was interrupted for two subjects in the Mg–vitamin B6 combination arm and four in the Mg arm due to AEs. All six subjects recovered from the AEs. One participant in the Mg arm experienced a SAE (severe gastroenteritis that led to hospitalization), and was subsequently withdrawn from the trial. This SAE was considered not to be related to trial medication as assessed by the trial physician; therefore, the participant received the treatment until trial withdrawal. No deaths occurred during the course of the trial.

## Discussion

This is the first randomized clinical trial to evaluate the effects of magnesium and vitamin B6 (Mg–vitamin B6) supplementation versus magnesium alone (Mg) on stress in individuals with stress and low serum magnesium concentrations using a validated psychometric measure of perceived stress (the DASS-42 stress subscale). Both treatments were administered in accordance with the recommended posology, relevant for the prevention and treatment of magnesium deficiency and associated symptoms, including stress-related symptoms like mild anxiety, and nervousness. The 300 mg elemental magnesium provided by the treatments covers 75–100% of the recommended daily intake [24], and a dosing regimen spread over two-to-three intakes per day is generally recommended to improve magnesium bioavailability [25]. Formulated as magnesium salt of organic acid (lactate), both Magne B6 and Magnespasmyl have greater solubility than magnesium salts of inorganic acids, and are therefore associated with better absorption and increased magnesium bioavailability [26–29]. Although previous results from experimental clinical trials were suggestive of a beneficial effect of the specific combination of magnesium lactate and vitamin B6 in a 10:1 ratio on subjective mood following several weeks of treatment [17], no randomized clinical trial has previously investigated the efficacy of such a formula on perceived stress in healthy adults.

Both interventions rapidly reduced stress from baseline, as indicated with a reduction in DASS-42 stress subscale score across both treatment arms of approximately 30% (~8 points) at Week 4 and 40% (~12 points) at Week 8 in the whole trial population (**Table 3**). Given that the mean (SD) DASS-42 stress subscale score across all subjects at baseline was 27.7 (7.1), this represents a clinically relevant reduction, being sufficient to move a participant with severe stress to the moderate category and a participant with moderate stress to the mild category. At Week 8, the stress reduction with Mg–vitamin B6 treatment was approaching 50% compared with baseline in severely stressed individuals. Numerous studies have confirmed the robust psychometric properties of the DASS-42 test in both adult and elderly populations with mood or anxiety disorders, with internal consistency ranging from 0.88 to 0.95 for the stress subscale [19, 30, 31]. The large magnitude of effect observed in this trial provides strong evidence for the benefits of Mg supplementation in stressed individuals with low serum magnesium; however, a placebo-controlled trial would be required to determine the true extent of benefits, especially given the large placebo effects observed in previous studies [17].

The benefits of magnesium in this population with low serum magensium concentration could be attributed to the effect of Mg levels on resistance to stress. Catecholamines and corticosteroids released during periods of stress decrease serum magnesium concentration through urinary excretion [3,5]. Conversely, low serum magnesium concentration increases the release of these same stress-associated hormones, leading to a positive feedback loop that enhances both the release of stress hormones and the depletion of magnesium [4,6].

Although superiority of Mg–vitamin B6 versus Mg was not demonstrated in the whole population, a statistically significant interaction was identified between baseline DASS-42 stress subscale score and treatment. According to recent regulatory guidance and EMA guidelines, the identification of a qualitative interaction that illustrates heterogeneous benefits within trial populations warrants further subgroup analyses [23, 32], thus a subgroup analysis was performed in line with the statistical analysis plan. Although a possible bias should be considered, due to the fact that randomization was performed on the initial whole population and not on the subgroups, this analysis revealed significantly greater reductions in the symptoms of stress with Mg–vitamin B6 than with Mg alone in subjects with severe and extremely severe stress, but no difference in those with normal to moderate stress. The majority of the improvements in DASS-42 stress subscale score occurred between baseline and Week 4 for both treatment arms, with incremental improvements seen between Week 4 and Week 8. Of note, in subjects with severe or extremely severe stress, Mg–vitamin B6 reduced stress levels at Week 4 to a similar extent (mITT, 37%; PP, 38%) (**Table 3**) as Mg alone at Week 8 (mITT and PP, 41.3%). These data suggest Mg–vitamin B6 relieved stress more rapidly during the treatment period compared with Mg alone, which may indicate a more rapid onset of action associated with Mg–vitamin B6 than with Mg alone. These results demonstrate that the combination of magnesium and vitamin B6 was 24% more effective in reducing stress compared with magnesium alone in this subgroup of severely stressed healthy adults. The beneficial effects of Mg–vitamin B6 in severely stressed healthy adults may be attributed to the complementary effects of Mg and vitamin B6, which have been demonstrated clinically in a number of studies using different psychometric and laboratory measures [14]. In a cohort of 9 healthy female volunteers, high-dose vitamin B6 (100 mg twice a day for four weeks) was shown to enhance Mg concentrations in plasma and red blood cells [15]. Clinical trials have clearly demonstrated the superiority of vitamin B6 (40–50 mg per day) in combination with Mg (200–250 mg per day) over Mg alone on subjective measures of anxiety and mild depression in women with premenstrual syndrome [33, 34], and the superiority of Mg–vitamin B6 over placebo in reducing anxiety [17]. This has led to the hypothesis that Mg–vitamin B6 influences anxiety states via moderation of the stress response [17], possibly by vitamin B6 facilitating cellular uptake of magnesium by limiting its excretion and increasing its effectiveness [15]. In addition, a prospective intervention trial evaluating Mg–vitamin B6 supplementation over 6 to 8 weeks reported improvements in autonomic nervous system function and perceived stress (as measured by the Ray-Holmes Life Events Scale) in women with stress and low serum magnesium concentrations [35]. Furthermore, the addition of vitamin B6 to a magnesium supplement could reduce the risk of homocysteinemia, thus providing additional benefits [36]. The results of the current trial add to this body of literature, and suggest that the complementary effects of Mg in combination with vitamin B6 are more pronounced in people with severe and extremely severe stress compared with moderately stressed individuals.

Everyday stress is a part of modern life and can be a major influencer on mood, sense of well-being, behavior and health [37]. Daily stressors have been shown to predict the emergence of both physical and mental health problems including influenza-like illness, sore throat, headaches and symptoms of depression and anxiety [38, 39]. Higher levels of perceived overall workplace stress correlate with greater degrees of both depressive and anxiety symptoms [40]. Chronic physical conditions linked to long-term negative reactivity to stressors include digestive, pain and urinary bladder disorders, which are in turn associated with psychological ramifications and high healthcare costs [41]. Approximately 60% of subjects in the current trial had severe to extremely severe stress (DASS-42 stress subscale score >25); thus the impact of stress on the lives of these individuals is likely to be high, and the benefits of the observed reductions in stress, if maintained over the long term, could have considerable positive effects on the physical and mental health of these individuals.

The safety profiles of both Mg–vitamin B6 and Mg were comparable and consistent with their respective labels (Magne B6 SmPC; Magnespasymyl SmPC) [16, 42]. The overall incidence of AEs and treatment-related AEs was slightly lower in the Mg–vitamin B6 arm compared with the Mg arm, and the most frequent AEs occurred at a similar frequency for both interventions. Only one participant (in the Mg arm) experienced a SAE, which was considered unrelated to treatment. No safety concerns related to the use of magnesium with or without vitamin B6 in stressed individuals with low serum magnesium concentration were highlighted by this trial.

In conclusion, both Mg–vitamin B6 and Mg alone reduced stress from baseline to Week 8 by approximately 40% in the overall adult population sample studied here, with no difference between arms. In people with severe or extremely severe stress with low serum magnesium concentration, the Mg–vitamin B6 combination provided a 24% greater reduction in stress than Mg alone at Week 8. These clinical data support the use of Mg supplementation to reduce stress in stressed adults with low serum magnesium concentrations. In addition, the results provide clinical support for a superior benefit of Mg combined with vitamin B6 in a 10:1 ratio (in the present study, Magne B6 SmPC) in adults with severe stress. Studies of longer duration are warranted to determine whether the effects seen can be maintained beyond 8 weeks.

## Supporting information

S1 Protocol Redacted study protocol. Redacted study protocol.
(PDF)

S2 Protocol Protocol publication approval form. Protocol publication approval.
(DOCX)

S1 Infographic An infographic illustrating the study and its overall findings. (PDF)

S1 Table CONSORT checklist. CONSORT checklist.
(DOC)
