# Supplementary material for: Superiority of magnesium and vitamin B6 over magnesium alone on severe stress in healthy adults with low magnesemia: A randomized, single-blind clinical trial
Source: PLoS One. 2018 Dec 18;13(12):e0208454. doi: 10.1371/journal.pone.0208454 (PMC6298677; doi:10.1371/journal.pone.0208454)
Supplement: S1 Protocol — Redacted study protocol. (PDF) [file pone.0208454.s001.pdf]

AMENDED CLINICAL TRIAL PROTOCOL 1

TITLE: Effects of MagneB6® (470.0 mg magnesium lactate dihydrate + 5.0 mg pyridoxine hydrochloride, coated tablet) supplementation (8 weeks) on stress levels of chronically stressed subjects, with suboptimal serum magnesium levels- a randomized, single-blind active comparator, multicentric clinical trial - comparison with Magnespasmyl® (465.4 mg magnesium lactate dihydrate, coated tablet)

COMPOUND: MagneB6®/ Magnesium lactate dihydrate + pyridoxine hydrochloride

STUDY NUMBER: MGLACC07810

STUDY NAME: MB6 SUPERIORITY

VERSION DATE / STATUS: 09-Feb-2016 / Approved

CLINICAL STUDY DIRECTOR: [redacted]

Protocol Amendment 1	Version number: 1	Date: 18-Feb-2016
Clinical Trial Protocol	Version number: 1	Date: 25-Nov-2015

EudraCT number: 2015-003749-24

Date: 18-Feb-2016 Total number of pages: 66

[redacted]
