# Supplementary material for: Superiority of magnesium and vitamin B6 over magnesium alone on severe stress in healthy adults with low magnesemia: A randomized, single-blind clinical trial
Source: PLoS One. 2018 Dec 18;13(12):e0208454. doi: 10.1371/journal.pone.0208454 (PMC6298677; doi:10.1371/journal.pone.0208454)
Supplement: S2 Protocol — Protocol publication approval. (DOCX) [file pone.0208454.s002.docx]

***Principles for Responsible Sharing of Clinical Trial Data, Information, and Results***

To the Editor of Plos ONE,

Henceforth, Sanofi approves publication under the Creative Commons Attribution (CC BY) 4.0 license, of the redacted amended clinical trial protocol titled “Effects of MagneB6® (470.0 mg magnesium lactate dihydrate + 5.0 mg pyridoxine hydrochloride, coated tablet) supplementation (8 weeks) on stress levels of chronically stressed subjects, with suboptimal serum magnesium levels- a randomized, single-blind active comparator, multicentric clinical trial - comparison with Magnespasmyl® (465.4 mg magnesium lactate dihydrate, coated tablet)” (dated 09-Feb-2016, EudraCT number: 2015-003749-24) as a supplementary material to the manuscript “Superiority of magnesium and vitamin B6 over magnesium alone on severe stress in healthy adults with low magnesemia: a randomized, single-blind clinical trial”.

Sanofi group
