# Supplementary material for: Superiority of magnesium and vitamin B6 over magnesium alone on severe stress in healthy adults with low magnesemia: A randomized, single-blind clinical trial
Source: PLoS One. 2018 Dec 18;13(12):e0208454. doi: 10.1371/journal.pone.0208454 (PMC6298677; doi:10.1371/journal.pone.0208454)
Supplement: S1 Infographic — (PDF) [file pone.0208454.s003.pdf]

SUPERIORITY OF MAGNESIUM AND VITAMIN B6 OVER MAGNESIUM ALONE ON SEVERE STRESS IN HEALTHY ADULTS WITH LOW MAGNESEMIA

A RANDOMIZED, SINGLE-BLIND, CLINICAL TRIAL

Primary Objective

To compare the effect of magnesium and vitamin B6 (Magne B_6®) supplementation vs magnesium supplementation alone on perceived stress

WHY was the trial carried out?

Work-related stress has been shown to affect up to **28%** of adults in the EU[1]

Physiological interactions between magnesium deficiencies and stress have been well-documented[2–4]

Mg Magnesium: **Serum magnesium** can moderate the response to **STRESS**

Vitamin B6: Modulates neurotransmitters that affect **DEPRESSION** and **ANXIETY**

Mg Magnesium + Vitamin B6: In combination may exhibit beneficial **SYNERGISTIC EFFECTS** on **SEVERE STRESS**

WHO took part in the trial?

- **268** Healthy adults
- **31.6** years Mean age
- **74%** Sex (female)
- **61%** With a DASS-42 stress subscale score of severe/extremely severe
- Mean serum magnesium **0.8** mmol/L
- Mean BMI **23.0** kg/m²

WHERE was the trial carried out?

4 locations in France

How was the trial carried out?

Phase IV clinical trial

- Randomized 1:1
- Stratified by sex
- Investigator blinded

Mg Magnesium 300 mg — 2 tablets 3 times a day with food

VS

Mg Magnesium 300 mg + Vitamin B6 30 mg — 2 tablets 3 times a day with food

8 weeks

How was stress measured?

Using the Depression Anxiety Stress Scale **(DASS-42)**,[5,6] a **CLINICALLY VALIDATED** self-reported measure of:

1. Depression
2. Anxiety
3. Stress

The **STRESS** component of DASS-42 comprises 14 questions with a 4-point scale of scoring

SCORING SCALE

0 = did not apply to me at all;
1 = applied to me to some degree, or some of the time;
2 = applied to me a considerable degree, or a good part of the time;
3 = applied to me very much, or most of the time

Stress and magnesium: a vicious circle

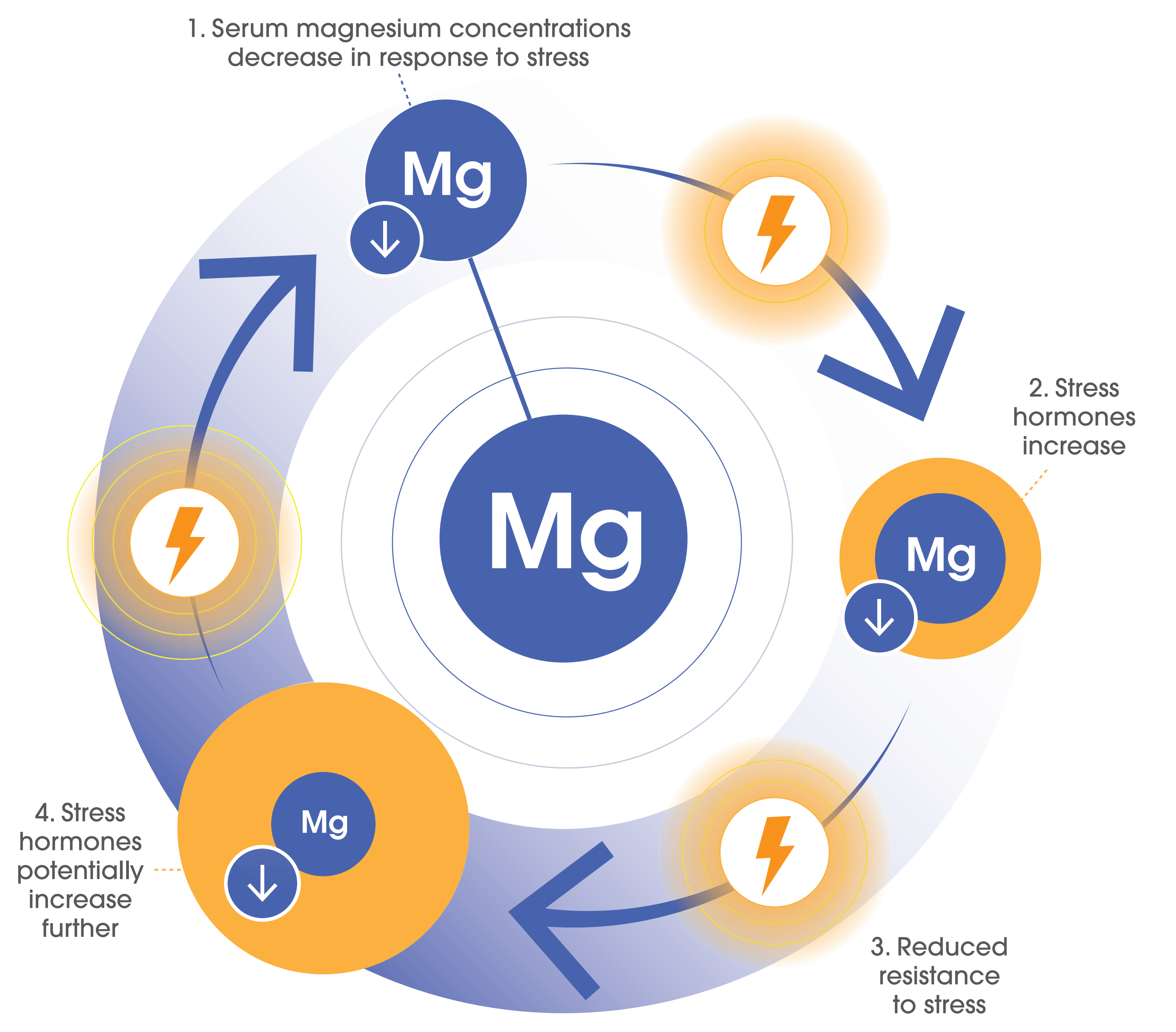

The trial DATA

WHAT were the results

Both interventions saw a **MARKED REDUCTION IN STRESS** (as measured by DASS-42) in the total (mITT) population* at Week 8†

Mg Magnesium: **42%** — 11.7 point improvement

Mg Magnesium + Vitamin B6: **45%** — 12.4 point improvement

Subgroup analyses§,**

Week 8

mITT n=162

Severe/extremely severe stress
DASS-42 stress subscale score 26–42

Mg B6: **16.4** point improvement
Mg: **13.2** point improvement

24% improvement on Mg Magnesium + Vitamin B6 VS Mg Magnesium
3.16 (95%CI: 0.50, 5.82; p=0.0203)

mITT n=102

Normal/moderate stress
DASS-42 stress subscale score 0–25

Mg B6: **8.2** point improvement
Mg: **10.6** point improvement

No significant difference observed between Mg Magnesium + Vitamin B6 VS Mg Magnesium
-2.36 (95%CI: -4.99, 0.27; p=0.0784)

PP n=160

Severe/extremely severe stress
DASS-42 stress subscale score 26–42

Mg B6: **16.1** point improvement
Mg: **13.2** point improvement

22% improvement on Mg Magnesium + Vitamin B6 VS Mg Magnesium
2.89 (95%CI: 0.22, 5.56; p=0.0339)

PP n=73

Moderate stress
DASS-42 stress subscale score 19–25

Mg B6: **9.7** point improvement
Mg: **11.7** point improvement

No significant difference observed between Mg Magnesium + Vitamin B6 VS Mg Magnesium
-1.96 (95%CI: -5.11, 1.20; p=0.2199)

Significantly greater improvements observed in **severely stressed** subjects

SAFETY

Both interventions had similar safety profiles

Mg Magnesium + Vitamin B6: 41% reported ≥1 adverse event; **12%** reported ≥1 adverse event possibly related to treatment

Mg Magnesium: 40% reported ≥1 adverse event; **17%** reported ≥1 adverse event possibly related to treatment

The most common treatment-related adverse events were...

Mg + Vitamin B6		Mg
**5%** diarrhea	Diarrhea	**8%** diarrhea
**2%** abdominal pain	Abdominal pain	**5%** abdominal pain

TO CONCLUDE...

Magnesium supplementation alone and Magnesium plus vitamin B6 supplementation reduced stress (by DASS-42) in all adults with low magnesemia

SEVERELY STRESSED individuals had the **GREATEST BENEFIT** with **MAGNESIUM PLUS VITAMIN B6 SUPPLEMENTATION**

Studies of longer duration are needed to determine **LONG-TERM EFFECTS**

EudraCT Number: 2015-003749-24. The trial was funded by Sanofi-Aventis Groupe, Gentilly, France, the manufacturer of Magne B_6®

*mITT, modified Intention-To-Treat population. Included all subjects with an evaluable DASS-42 stress subscale score at baseline and at least one other time point during the treatment period.

†Similar results were observed at Week 4 and in the PP population.‡

‡PP, Per Protocol Set. Included all participants in the mITT without any major protocol deviations. The PP excluded patients with baseline DASS-42 stress subscale scores ≤18.

§Similar results were observed at Week 4.

**In conjunction with EMA guidelines,[7] a predetermined subgroup analysis was carried out following the identification of heterogeneous benefits demonstrated within the treatment groups.

References
1. Third European survey on working conditions. 2000; https://www.eurofound.europa.eu/sites/default/files/ef_publication/field_ef_document/ef0121en.pdf. Accessed August 2018.
2. Jung K-I, Ock SM, Chung JH, Song CH. Associations of serum Ca and Mg levels with mental health in adult women without psychiatric disorders. Biol Trace Elem Res 2010;133:153.
3. Abbasi B, Kimiagar M, Sadeghniiat K, Shirazi MM, Hedayati M, Rashidkhani B. The effect of magnesium supplementation on primary insomnia in elderly: A double-blind placebo-controlled clinical trial. J Res Med Sci 2012;17(12):1161–1169.
4. Zogovic D, Pesic V, Dmitrasinovic G, et al. Pituitary-gonadal, pituitary-adrenocortical hormones and IL-6 levels following long-term magnesium supplementation in male students. J Med Biochem 2014;33(3):291–298.
5. Brown TA, Chorpita BF, Korotitsch W, Barlow DH. Psychometric properties of the Depression Anxiety Stress Scales (DASS) in clinical samples. Behav Res Ther 1997;35(1):79–89.
6. Lovibond, SH, Lovibond, PF. Manual for the Depression Anxiety Stress Scales. 2nd ed. Sydney: Psychology Foundation.
7. Committee for Proprietary Medicinal Products (CHMP). Points to consider on adjustment for baseline covariates. 2003. http://www.ema.europa.eu/docs/en_GB/document_library/Scientific_guideline/2009/09/WC500003639.pdf. Accessed April 2018.
